# Lysophosphatidic acid increases mesangial cell proliferation in models of diabetic nephropathy via Rac1/MAPK/KLF5 signaling

**DOI:** 10.1038/s12276-019-0217-3

**Published:** 2019-02-15

**Authors:** Donghee Kim, Hui Ying Li, Jong Han Lee, Yoon Sin Oh, Hee-Sook Jun

**Affiliations:** 1 Lee Gil Ya Cancer and Diabetes Institute, Gachon University, Incheon, Republic of Korea; 2 Department of Nephrology, Affiliated Hospital of Yanbian University, Yanbian, China; 3 College of Pharmacy, Gachon University, Incheon, Republic of Korea; 4 Department of Food and Nutrition, Eulji University, Seongnam, Republic of Korea; 5 Gachon Medical and Convergence Institute, Gachon Gil Medical Center, Incheon, Republic of Korea

**Keywords:** Diabetes complications, Mechanisms of disease

## Abstract

Mesangial cell proliferation has been identified as a major factor contributing to glomerulosclerosis, which is a typical symptom of diabetic nephropathy (DN). Lysophosphatidic acid (LPA) levels are increased in the glomerulus of the kidney in diabetic mice. LPA is a critical regulator that induces mesangial cell proliferation; however, its effect and molecular mechanisms remain unknown. The proportion of α-SMA^+^/PCNA^+^ cells was increased in the kidney cortex of *db/db* mice compared with control mice. Treatment with LPA concomitantly increased the proliferation of mouse mesangial cells (SV40 MES13) and the expression of cyclin D1 and CDK4. On the other hand, the expression of p27^Kip1^ was decreased. The expression of Krüppel-like factor 5 (KLF5) was upregulated in the kidney cortex of *db/db* mice and LPA-treated SV40 MES13 cells. RNAi-mediated silencing of KLF5 reversed these effects and inhibited the proliferation of LPA-treated cells. Mitogen-activated protein kinases (MAPKs) were activated, and the expression of early growth response 1 (Egr1) was subsequently increased in LPA-treated SV40 MES13 cells and the kidney cortex of *db/db* mice. Moreover, LPA significantly increased the activity of the Ras-related C3 botulinum toxin substrate (Rac1) GTPase in SV40 MES13 cells, and the dominant-negative form of Rac1 partially inhibited the phosphorylation of p38 and upregulation of Egr1 and KLF5 induced by LPA. LPA-induced hyperproliferation was attenuated by the inhibition of Rac1 activity. Based on these results, the Rac1/MAPK/KLF5 signaling pathway was one of the mechanisms by which LPA induced mesangial cell proliferation in DN models.

## Kidney disease: Mechanisms of diabetes-related damage

A potent molecular mediator of diabetic kidney disease induces its pathogenic effects via proteins that could be targeted with future drug therapies. Yoon Sin Oh from Eulji University in Seongnam-si and Hee-Sook Jun from Gachon University in Incheon, both in South Korea, and colleagues treated certain cells found in the kidney’s glomerulus, the organ’s filtering unit, with a signaling molecule called lysophosphatidic acid (LPA) that is elevated in the blood of diabetic mice. They showed that LPA stimulated cellular proliferation and boosted the expression of proteins involved in regulating the cell cycle and a multipurpose signaling pathway. They then inhibited the activity of these proteins to prevent the kidney cells’ hyperproliferation, both in cell culture and in diabetic mice. The results highlight the potential of blocking mediators of LPA signaling to treat kidney-related complications of diabetes.

## Introduction

Diabetic nephropathy (DN) is a well-known microvascular complication in patients with diabetes and a common cause of end-stage renal disease worldwide, contributing to the overall morbidity and mortality of patients with diabetes^1,2^.

Glomerular mesangial cells, one of the major types of resident renal cells, are involved in the processes of DN. Mesangial cell proliferation is stimulated in the early stage during the progression of the disease; subsequently, the growth of the cells is arrested and cells undergo hypertrophy^3^. Therefore, the increased proliferation of mesangial cells is a crucial contributor to the initial pathophysiological mechanism in early-stage DN, which eventually causes chronic renal failure^4^. Various pathogenic factors, including hyperglycemia, dyslipidemia, hypertension, and the accumulation of advanced glycation end products (AGEs), promote mesangial cell proliferation, leading to the accumulation of extracellular matrix proteins and thickening of the glomerular basement membrane^4–6^. Therefore, the inhibition of mesangial cell proliferation is one of the strategies used to control DN progression in the initial stages.

Lysophosphatidic acid (LPA) is a small glycerophospholipid that regulates different cellular responses, such as proliferation, survival, and migration, via G protein-coupled receptors (GPCRs; LPA receptors 1–6)^7^. LPA induces the proliferation of different types of cells^8–11^. However, its effect on mesangial cell proliferation during the development of DN remains unclear.

Previous studies have reported a marked increase in LPA levels in the glomeruli of diabetic mice^12^ and high-fat diet-induced obese mice^13^. Moreover, LPA induces fibrosis in SV40 MES13 cells, and the inhibition of LPA receptor 1 (LPAR1) signaling ameliorates DN in diabetic *db/db* mice^14^. These findings suggest the involvement of LPA in the hyperproliferation of renal cells. We sought to determine the underlying mechanisms to obtain a better understanding of the pathophysiology of the initial stage of DN using an animal model of type 2 diabetes and an in vitro model.

In this study, LPA stimulated the proliferation of renal mesangial cells via cell cycle regulatory proteins. Moreover, the Ras-related C3 botulinum toxin substrate 1/mitogen-activated protein kinase/Krüppel-like factor 5 (Rac1/MAPK/KLF5) signaling pathway may be involved in the pro-proliferative effect of LPA during the development of DN.

## Materials and methods

### Cell culture

Mes13 cells from a SV40 transgenic mouse (SV40 MES13) were maintained in Dulbecco’s modified Eagle’s medium (Welgene Inc., Daegu, South Korea) containing 5% fetal bovine serum (Life Technologies, Grand Island, NY, USA) and 1% penicillin–streptomycin (Welgene Inc.). Cells were plated in a six-well plate (2 × 10^5^ cells/well) to investigate the effect of LPA on SV40 MES13 cells. After 12 h, cells were pretreated with serum-free medium containing 0.1% fatty acid-free bovine serum albumin (Sigma-Aldrich, St. Louis, MO, USA) for 12–16 h. Subsequently, the cells were treated with LPA (Avanti POLAR LIPIDS, Alabaster, AL, USA).

### Animals

Nine-week-old male diabetic *db/db* (BKS.Cg-lepr^db^/lepr^db^) mice on the C57BLKS/J background were obtained from Korea Research Institute of Bioscience and Biotechnology (KRIBB, Daejeon, South Korea)^15,16^. Age-matched, nondiabetic wild-type (BKS.Cg-lepr^+^/lepr^+^, WT) mice were used as the control group. All experiments were approved by the Institutional Animal Care and Use Committee of Gachon University.

### Histological analysis of the kidneys

The mice were killed and their kidneys were removed. The right kidney was fixed with neutral buffered formalin (10%, Sigma-Aldrich), embedded in paraffin, and sectioned at 5 μm. For immunofluorescence staining, kidney sections were stained with rabbit anti-proliferating cell nuclear antigen (PCNA) (Cell Signaling Technology, Inc., Danvers, MA, USA) and mouse anti-α-smooth muscle actin (α-SMA) (Abcam, Cambridge, UK) primary antibodies, Alexa Fluor^®^ 488-conjugated anti-rabbit (Abcam) and DyLight^®^ 550-conjugated anti-mouse (Bethyl Laboratories, Inc., Montgomery, TX, USA) secondary antibodies, and 4′-6-diamidino-2-phenylindole (DAPI, Invitrogen Molecular Probes, Carlsbad, CA, USA). Furthermore, 30 glomeruli per mouse (*n* = 3–4) were examined under a confocal microscope (LSM 700; Carl Zeiss Inc., Oberkochen, Germany).

### Cell proliferation assay

SV40 MES13 cell proliferation was examined using a Cell Counting Kit-8 (CCK-8; Dojindo, Kumamoto, Japan) according to the manufacturer’s protocol. Approximately 0.8 × 10^4^ cells/well were plated in a 96-well plate. After treatment with LPA, 10 μl of CCK-8 solution were added to each well and incubated for 3 h. Cell viability was measured by determining the optical density at 450 nm (VersaMax Microplate Reader, Molecular Devices, LLC, Sunnyvale, CA, USA).

### Transfection

SV40 MES13 cells were transfected with the empty vector and pcDNA3-EGFP-Rac1-T17N (dominant-negative form of Rac1; Addgene, Cambridge, MA, USA) plasmid DNA using the Lipofectamine 2000 reagent (Invitrogen) according to the manufacturer’s instructions. For small interfering RNA transfection, SV40 MES13 cells were plated and transiently transfected with 75 pM of KLF5 siRNA or scrambled siRNA (Santa Cruz Biotechnology, Santa Cruz, CA, USA) using the Lipofectamine RNAiMAX (Invitrogen) reagent according to the manufacturer’s instructions. After 36 h, the medium was replaced with serum-free medium containing 0.1% fatty acid-free bovine serum albumin, incubated for 12–16 h, and then cells were stimulated with LPA (10 μM) for various times.

### Analysis of the activity of Rho family GTPases

RhoA, Rac1, and Cdc42 activity assays were performed using the appropriate assay kits according to the manufacturer’s instructions (Cell Biolabs, San Diego, CA, USA). The relative levels of active GTPases were determined by measuring the amount of GTPases sedimented by the GST-Rac1/Cdc42-binding domain of p21-activated kinase 1 or the GST-RhoA-binding domain of rhotekin relative to the amount in whole-cell lysates. Bound proteins were resolved on 15% SDS-PAGE gels and immunoblotted with anti-RhoA (1:1000), anti-Rac1 (1:500), or anti-Cdc42 (1:1000) antibodies (Cell Biolabs).

### Quantitative real-time PCR (qRT-PCR)

The total RNA was prepared using RNAiso Plus (TaKaRa, Shiga, Japan), and cDNAs were synthesized from 5 µg of RNA using a PrimeScript 1st strand cDNA Synthesis Kit (TaKaRa). qPCR was performed with SYBR^®^ Premix Ex Taq^TM^ II, ROX plus (TaKaRa) using the ABI 7900 Real-Time PCR system (Applied Biosystems, Foster City, CA, USA), according to the protocols provided by the manufacturer. The relative levels of each mRNA transcript were calculated using the 2Δ^–CT^ method, in which Δ^CT^ represents the difference in threshold cycle values between the target mRNA and the cyclophilin internal control. Primers used for qRT-PCR are listed in Table 1.

**Table 1:** Primers for qRT-PCR

Gene	Forward	Reverse
Cyclophilin	TGGAGAGCACCAAGACAGACA	TGCCGGAGTCGACAATGAT
KLF5	CACCCCACCTCCGTCCTAT	GGGTTGTGAATCGCCAGTTT
Egr1	CACTCACCCACCATGGACAA	CCCGTTGCTCAGCAGCAT
p27^Kip1^	TCTTCGGCCCGGTCAA	CCGGCAGTGCTTCTCCAA

### Western blot analysis

Kidney tissues and cells were lysed in mammalian protein extraction buffer (GE Healthcare, Milwaukee, WI, USA) containing a protease and phosphatase inhibitor cocktail (GenDEPOT Inc., Katy, TX, USA). Approximately 20–50 µg of the lysates were electrophoresed and transferred to polyvinylidene difluoride membranes (PVDF, Millipore, Billerica, MA, USA). Membranes were blocked with 3% nonfat milk and incubated with primary antibodies overnight at 4 °C. The following antibodies were used at the indicated dilutions: anti-cyclin A, anti-cyclin D1, anti-CDK2, anti-CDK4, anti-p27^Kip1^, anti-KLF5, anti-β-actin (1:500; Santa Cruz Biotechnology), anti-p-Erk, anti-Erk, anti-p-JNK, anti-JNK, anti-p-p38, anti-p38, and Egr1 (1:1000; Cell Signaling Technology). After washes, the membranes were incubated with secondary antibodies conjugated with horseradish peroxidase (Santa Cruz Biotechnology) for 1 h at room temperature. Immunoreactive signals were detected using an ECL detection system (Millipore).

### Statistical analyses

The results are presented as means ± standard error of the mean (SEM). A two-tailed unpaired *t* test was used to analyze differences between two groups with GraphPad Prism software. Differences between more than two groups were analyzed using one-way ANOVA with SPSS software. A *p*-value of < 0.05 was considered significant.

## Results

### LPA induces mesangial cell proliferation

**Fig. 1:**
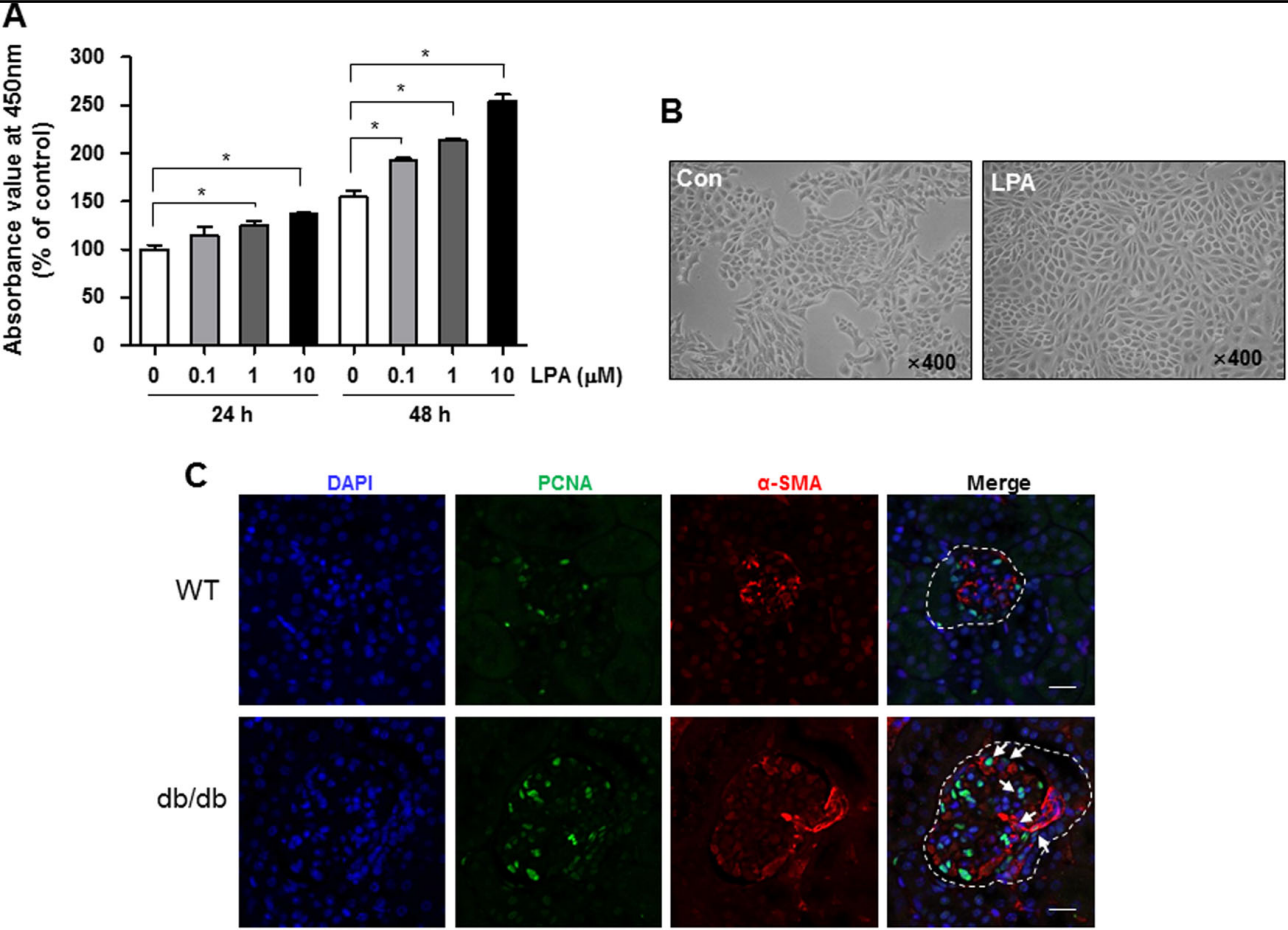
LPA increases SV40 MES13 cell proliferation. SV40 MES13 cells were plated and starved in serum-free medium containing 0.1% fatty acid-free bovine serum albumin. **a** Cells were treated with LPA at a final concentration of 0.1, 1, or 10 μM for 24 or 48 h. Cell proliferation was examined using the CCK-8 assay (*n* = 3 independent experiments). **b** The morphology of control (Con) and LPA-treated (10 μM) cells was examined using light microscopy (original magnification, × 400). **c** Representative images showing the colocalization of α-SMA (red) and PCNA (green) in kidney sections from 9-week-old *db/db* mice and age-matched wild-type (WT) mice. Nuclei were counterstained with DAPI (blue). Dashed line, kidney glomeruli; arrows, costained cells; scale bars, 20 μm; *n* = 3–4; *p < 0.05. Data are presented as means ± SEM

SV40 MES13 cells were treated with different doses (0.1, 1, or 10 μM) of LPA, and the viability was measured using CCK-8 assay to investigate whether LPA induces the proliferation of these cells. As shown in Fig. 1a, the number of viable cells was significantly increased after treatment with 1 μM LPA treatment for 24 h, and the proliferative effect of LPA was maintained for 48 h in a dose-dependent manner (Fig. 1a). Morphological changes and high-density cell growth were clearly observed under the microscope after treatment with 10 μM LPA for 24 h (Fig. 1b). Because LPA is expressed at high levels in the glomeruli of diabetic mice^12^, we examined the proliferation of cells in the kidney cortex of wild-type and diabetic *db/db* mice. We performed immunofluorescence staining of kidney sections with antibodies against α-SMA, which is a marker of mesangial cells, and PCNA. The number of α-SMA-positive cells was increased in the glomeruli of *db/db* mice compared with wild-type mice, and the number of cells double-stained with α-SMA/PCNA was also increased in the kidney cortex of *db/db* mice (Fig. 1c).

### LPA increases cyclin D1 and CDK4 expression and suppresses p27^Kip1^ expression

**Fig. 2:**
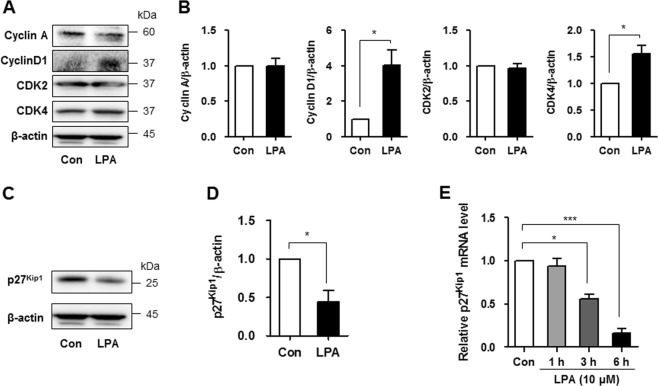
LPA increases cyclin D1 and CDK4 expression by suppressing p27^Kip1^ expression. SV40 MES13 cells were seeded in a six-well plate and starved in serum-free medium containing 0.1% fatty acid-free bovine serum albumin for 12–16 h. **a** Cells were treated with LPA (10 μM) for 12 h, and the levels of the cyclin A, cyclin D1, CDK2, and CDK4 proteins were measured using western blot analyses (*n* = 3 independent experiments). **b** The relative levels of the proteins were normalized to β-actin and quantified using ImageJ software. **c** Cells were treated with LPA (10 μM) for 12 h, and the level of the p27^Kip1^ protein was measured using western blotting (*n* = 3 independent experiments). **d** The relative level of p27^Kip1^ was normalized to β-actin and quantified using ImageJ software. **e** Cells were treated with LPA (10 μM) for 1, 3, or 6 h, and the expression of the p27^Kip1^ mRNA was analyzed using qRT-PCR (*n* = 3 independent experiments). *p < 0.05, ***p < 0.001. Data are presented as means ± SEM

We examined the expression of various cyclins and cyclin-dependent kinases (CDKs) to investigate whether the cell cycle process is involved in the proliferation of LPA-treated SV40 MES13 cells. As cyclin A, cyclin D1, and related CDKs have been reported to be involved in the proliferation of mesangial cells^17^, we measured the levels of these proteins in cells treated with 10 μM LPA. No changes in cyclin A and CDK2 levels were observed; however, the levels of the cyclin D1 and CDK4 proteins were significantly increased 12 h after the LPA treatment (Fig. 2a). The quantitative analysis also revealed significantly increased cyclin D1/actin and CDK4/actin ratios in LPA-treated cells compared with control cells (Fig. 2b). CDK inhibitors bind to cyclin–CDK complexes, leading to cell cycle arrest^3,18^. Therefore, we measured the levels of various CDK inhibitors, such as p15^INK4b^, p16^INK4a^, p21^Cip1^, and p27^Kip1^. Levels of the p27^Kip1^ mRNA and proteins were significantly reduced in LPA-treated cells compared with control cells, but the levels of other CDK inhibitors were not changed (Fig. 2c, d, and Supplementary Figure 1). Levels of the p27^Kip1^ mRNA were measured at 1, 3, and 6 h after LPA treatment to investigate whether LPA decreased the expression of this gene in a time-dependent manner. As shown in Fig. 2e, the level of the p27^Kip1^ mRNA was significantly decreased after 3 h of LPA treatment (Fig. 2e).

### LPA upregulates KLF5 expression in mesangial cells

**Fig. 3:**
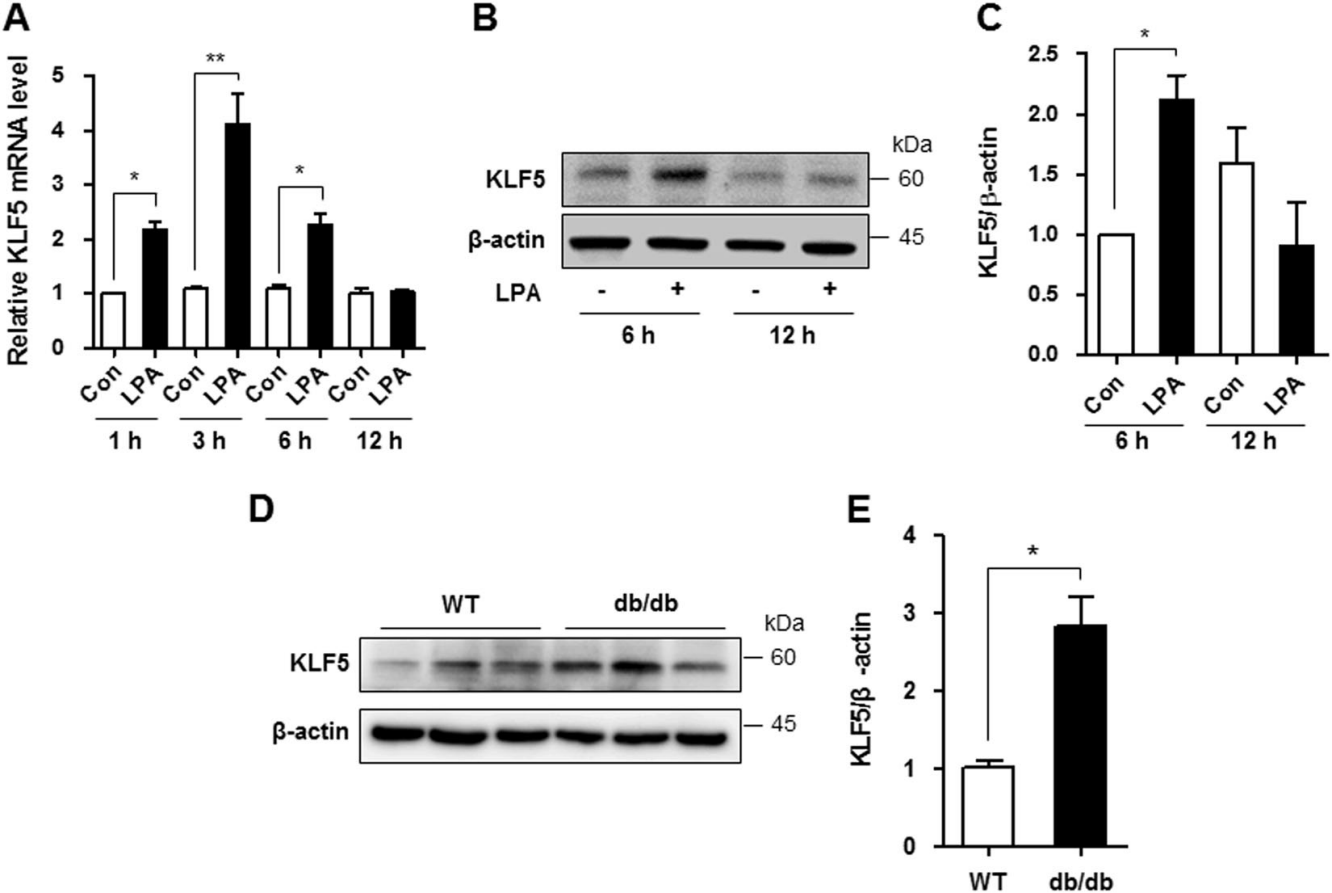
LPA upregulates KLF5 expression in SV40 MES13 cells. **a** The expression of the KLF5 mRNA at 1, 3, 6, or 12 h in cells treated with 10 μM LPA was analyzed using qRT-PCR (*n* = 3 independent experiments). **b** Western blots showing KLF5 and β-actin levels at 6 or 12 h after treatment with 10 μM LPA. **c** The relative level of KLF5 was normalized to β-actin and quantified using ImageJ software. **d** Western blot showing the level of the KLF5 protein in the renal cortex of 9-week-old wild-type (WT) and *db/db* mice. **e** The results were quantified, and β-actin was used as a loading control (*n* = 3–4). *p < 0.05, **p < 0.01. Data are presented as means ± SEM

KLF5 has been reported to negatively regulate p27^Kip1^ transcription in cancer cells^19,20^. As the role of KLF5 in DN is unknown, we examined the involvement of KLF5 in downregulating p27^Kip1^ expression in LPA-treated mesangial cells. As shown in Fig. 3a, the level of the KLF5 mRNA was significantly increased after 1, 3, and 6 h of LPA treatment (Fig. 3a), and the level of the KLF5 protein was significantly increased after 6 h of LPA treatment (Fig. 3b, c). In addition, significantly higher levels of the KLF5 protein were detected in the renal cortex of *db/db* mice than in wild-type mice (Fig. 3d, e), consistent with the findings from LPA-treated SV40 MES13 cells.

### Downregulation of KLF5 inhibits the LPA-induced proliferation of mesangial cells

**Fig. 4:**
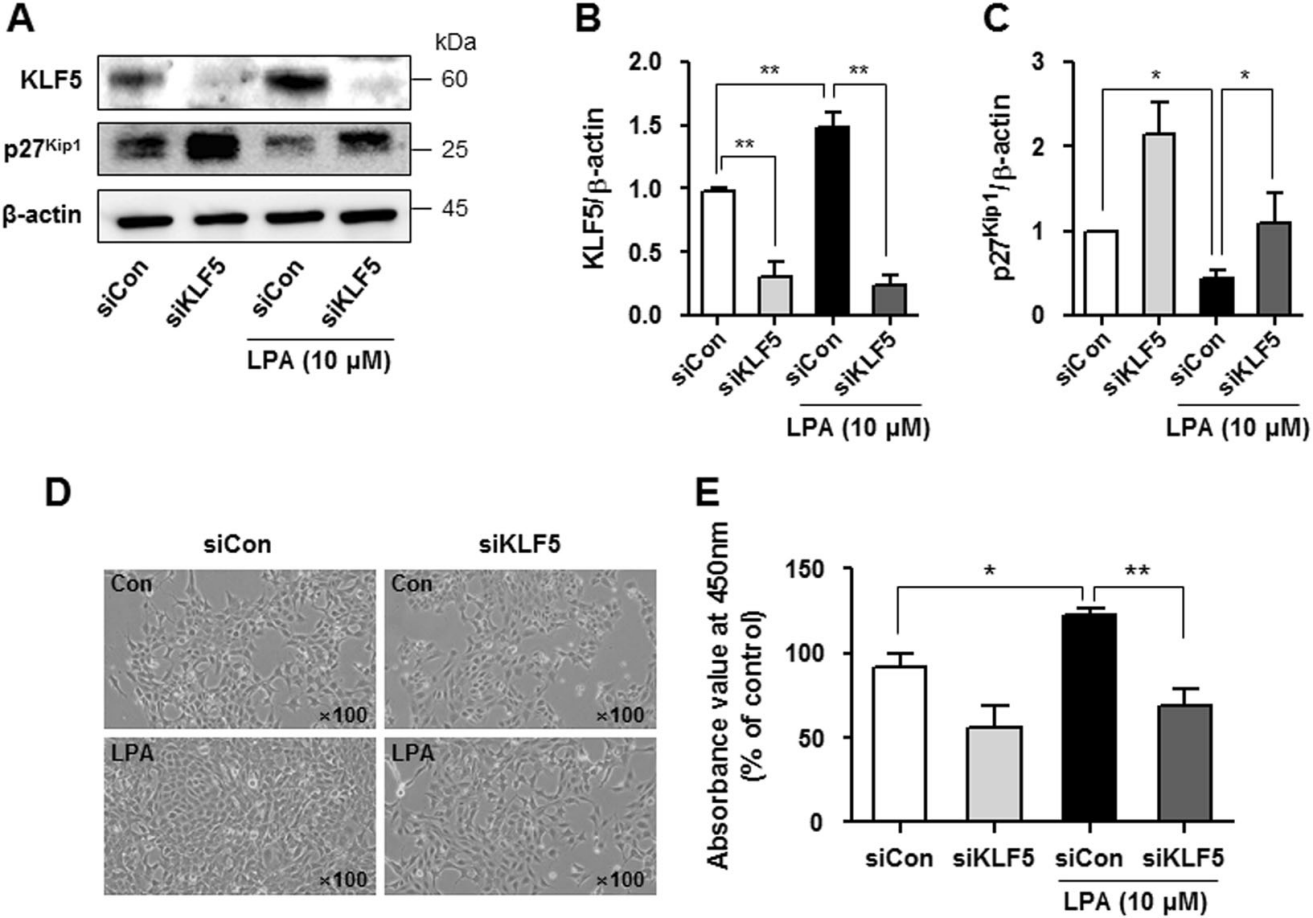
Downregulation of KLF5 inhibits the LPA-induced proliferation of SV40 MES13 cells. **a** Cells were transfected with a scrambled siRNA (siCon) or KLF5 siRNA (siKLF5) for 48 h and treated with LPA (10 μM) for 6 h, and the levels of the KLF5 and p27^Kip1^ proteins were determined using western blotting. **b**, **c** The relative levels of the proteins were normalized to β-actin and quantified using ImageJ software (*n* = 3 independent experiments). **d** Cell morphology was examined using light microscopy (original magnification, × 100), and (**e**) cell proliferation was analyzed using CCK-8 assay after treatment with 10 μM LPA and transfection of the KLF5 siRNA or scrambled siRNA for 24 h (*n* = 3 independent experiments). *p < 0.05, **p < 0.01. Data are presented as means ± SEM

We transfected SV40 MES13 cells with a siRNA targeting KLF5 and cultured them in the presence or absence of LPA to determine whether increased KLF5 expression downregulated p27^Kip1^ expression and increased mesangial cell proliferation. KLF5 expression was significantly reduced 48 h after KLF5 siRNA transfection (Fig. 4a, b), and LPA-induced p27^Kip1^ downregulation was inhibited in KLF5 siRNA-transfected cells compared with scrambled siRNA-transfected cells (Fig. 4c). Moreover, KLF5 knockdown significantly attenuated the LPA-induced proliferation of SV40 MES13 cells (Fig. 4d, e).

### LPA increases MAPK activation and Egr1 expression in mesangial cells

**Fig. 5:**
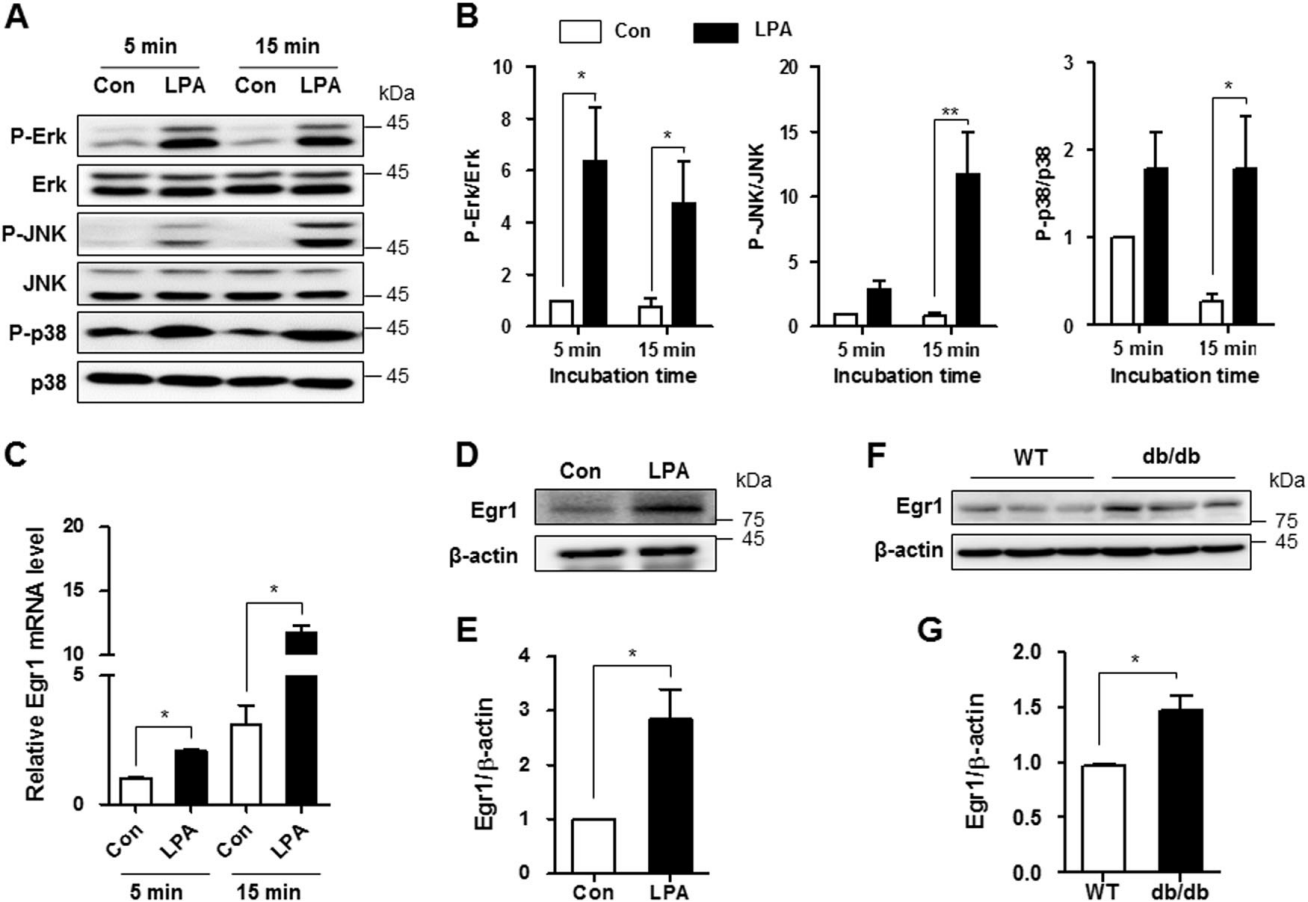
LPA increases MAPK activation and Egr1 expression in SV40 MES13 cells. **a** Western blots showing levels of MAPK proteins (p-Erk, total Erk, p-JNK, total JNK, p-p38, and total p38) in cells treated with 10 μM LPA for 5 or 15 min. **b** The relative protein levels were normalized to β-actin and quantified using ImageJ software (*n* = 3 independent experiments). **c** The expression of the Egr1 mRNA was analyzed using qRT-PCR in cells treated with 10 μM LPA for 5 or 15 min (*n* = 3 independent experiments). **d** Western blots showing the levels of the Egr1 and β-actin proteins in cells treated with 10 μM LPA for 6 h. **e** The relative Egr1 levels were normalized to β-actin and quantified using ImageJ software (*n* = 3 independent experiments). **f** Western blot showing the levels of the Egr1 protein in the renal cortex of 9-week-old wild-type (WT) and *db/db* mice. **g** The results were quantified, and β-actin was used as a loading control (*n* = 3–4). *p < 0.05, **p < 0.01. Data are presented as means ± SEM

The KLF5 promoter has an early growth response 1 (Egr1)-like sequence-binding site, and Egr1 expression is regulated by MAPK activation^21–23^. Therefore, we investigated whether LPA activated these signaling pathways in SV40 MES13 cells. SV40 MES13 cells were incubated with 10 μM LPA for 5 or 15 min, and the phosphorylation of Erk, JNK, and p38 was examined using western blot analysis. The levels of p-Erk were increased at 5 min and were maintained for 15 min after LPA stimulation. In addition, JNK and p38 were phosphorylated at 15 min after LPA treatment (Fig. 5a, b). The levels of the Egr1 mRNA and proteins were also significantly increased in LPA-treated cells compared with nontreated cells (Fig. 5c–e). Moreover, higher levels of the Egr1 were detected in the kidney cortex of *db/db* mice compared with the levels in control mice (Fig. 5f, g).

### LPA increases SV40 MES13 cell proliferation by activating the Rho GTPase Rac1

**Fig. 6:**
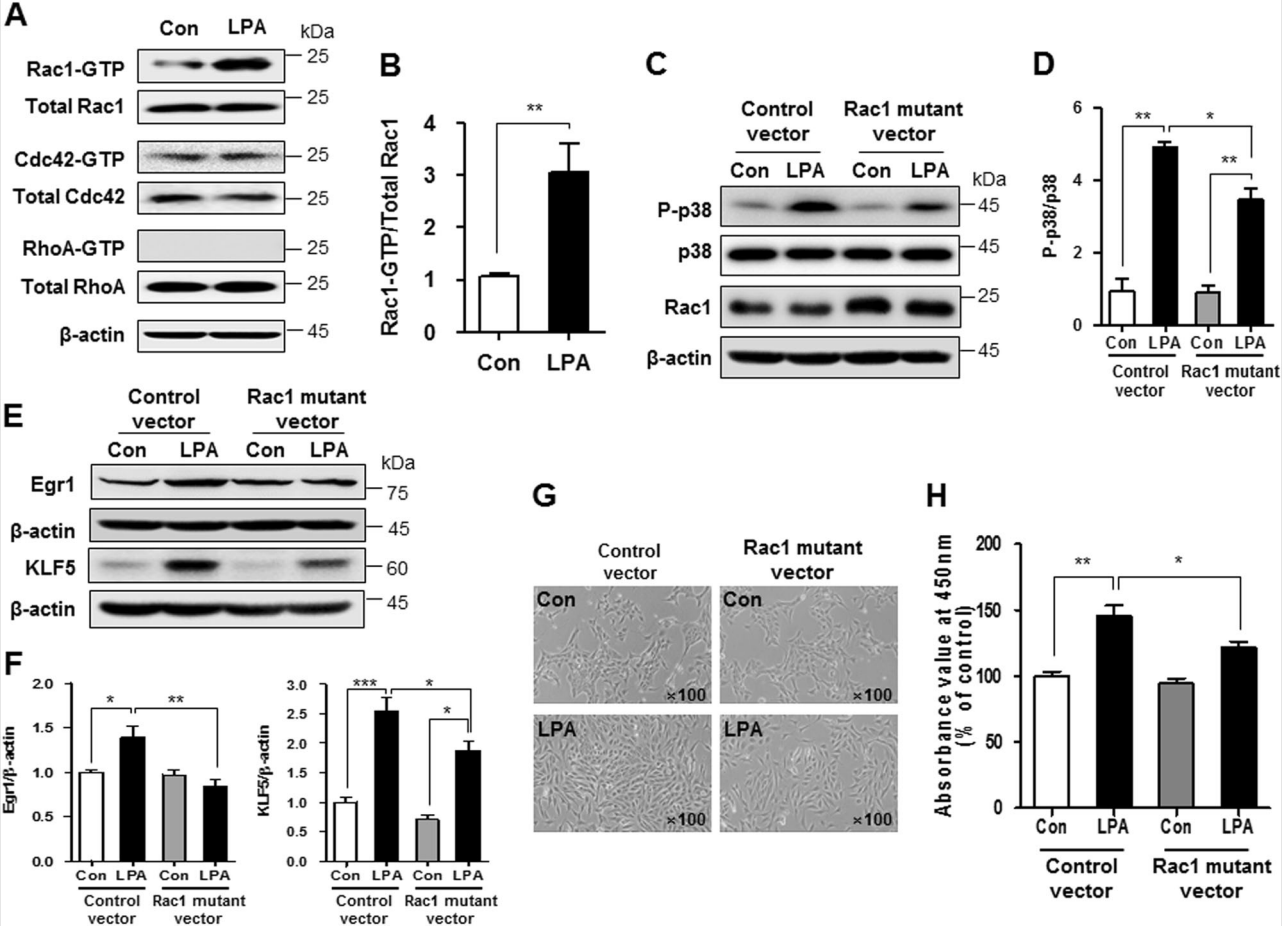
LPA increases SV40 MES13 cell proliferation by activating the Rho GTPase Rac1. **a** Western blots showing the levels of the RhoA, Rac1, and Cdc42 proteins in cells treated with 10 μM LPA for 5 min. RhoA, Rac1, and Cdc42 activity assays were performed using a GST-fusion protein derived from p21-activated kinase or rhotekin, which selectively binds to GTP-bound RhoA, Rac1, or Cdc42. GST–rhotekin precipitates were analyzed using western blotting. **b** The relative levels of active Rac1 were normalized to the levels of total Rac1 and quantified using ImageJ software (*n* = 3 independent experiments). **c**, **e** After the transfection with the control vector or the dominant-negative Rac1 mutant, SV40 MES13 cells were treated with LPA (10 μM) for 15 min (C) or 6 h (E). Western blots show the levels of p-p38, p38, Rac1, Egr1, KLF5, and β-actin proteins. **d**, **f** The relative levels of p-p38, Egr1, and KLF5 were normalized to p38 or β-actin and quantified using ImageJ software (n = 3–4 independent experiments). **g** Cell morphology was examined using light microscopy (original magnification, × 100), and (**h**) cell proliferation was examined using the CCK-8 assay (*n* = 3 independent experiments). *p < 0.05, **p < 0.01, ***p < 0.001. Data are presented as means ± SEM

Rho family GTPases, such as Ras homolog family member A (RhoA), Rac1, and cell division control protein 42 homolog (Cdc42), play critical roles in cell cycle regulation, cytoskeletal reorganization, and cell survival^24,25^. GTPase activity in control and LPA-treated cell lysates was analyzed using glutathione S-transferase (GST)–p21-activated kinase and GST–rhotekin to investigate the involvement of GTPases in LPA-induced cell proliferation. As shown in Fig. 6a, the levels of Rac1 and Cdc42 were not different; however, LPA significantly increased the GTPase activity of Rac1, but not Cdc42. Activation of the RhoA GTPase was not observed in LPA-treated cells, despite the high level of protein expression (Fig. 6a, b). We transfected dominant-negative mutants of Rac1 into SV40 MES13 cells to confirm the role of Rac1 in LPA-induced proliferation. First, we examined the inactivation of p38, Erk, and JNK in Rac1 mutant-transfected cells to determine which MAPK proteins were affected by LPA-induced Rac1 activation. As shown in Fig. 6c, d and Supplementary Figure 2, the levels of p-p38, but not p-Erk and p-JNK, were significantly reduced in the Rac1 mutant-transfected cells compared with the control vector-transfected cells. Moreover, the dominant-negative mutants of Rac1 partially but significantly attenuated the LPA-induced upregulation of Egr1 and KLF5 (Fig. 6e and f). Finally, the inhibition of Rac1 activity reduced LPA-induced cell proliferation compared with control vector-treated cells (Fig. 6g, h).

## Discussion

Mesangial expansion caused by cell hyperproliferation decreases the glomerular filtration rate and occurs in patients with DN before the onset of clinical manifestations^26^. Therefore, the inhibition of mesangial cell proliferation is considered one of the approaches to prevent and ameliorate DN. Certain growth-inducing factors (e.g., glucose and angiotensin II) and cytokines (e.g., transforming growth factor β) induce mesangial cell hyperproliferation and/or hypertrophy^27^. However, additional studies on the hyperproliferative factors and the molecular mechanisms involved are needed to develop the most efficient and effective treatment strategy for DN. According to several studies^9,10,28^, including the present study, LPA exerts a pro-proliferative effect on renal mesangial cells. Moreover, in our previous study, the kidney cortex of diabetic mice showed proliferative activity with high levels of LPA^12^. Thus, LPA might be an important proliferative factor that could induce glomerular injury during the process of DN.

The cell cycle is regulated by the balance between the activities or levels of cyclin-related proteins, CDK, and CDK inhibitors, which provide a mechanism for growth inhibition^18^. Cyclin D1 and CDK4 have been implicated in the mechanism controlling cell proliferation in the G1/S phase, and their overexpression promotes cell division and results in neoplastic transformation^29^. During this process, p27^Kip1^ tightly binds to the cyclin D1–CDK4 complex and induces cell cycle arrest^30,31^. Therefore, in addition to the decreased level of p27^Kip1^, the increased expression of cyclin D1 and CDK4 induced by the LPA treatment would promote the proliferation of mesangial cells. According to Liu et al., the expression of p27^Kip1^ is downregulated in rat mesangial cells stimulated with high glucose concentrations^32^, and some drugs that upregulate p27^Kip1^ expression exhibit potent anti-proliferative activity in mesangial cells^33,34^. However, Wolf et al. reported that chronic treatment (72–96 h) with high glucose concentrations in mouse mesangial cells increased glomerular levels of the p27^Kip1^ protein but not the p27^Kip1^ mRNA^35^. Mesangial cells show a biphasic growth response under high glucose conditions, with an initial proliferation phase followed by sustained hypertrophy associated with increased levels of extracellular matrix proteins^3^. During this process, the upregulation of p27^Kip1^ expression plays an important role in the transition from a proliferative to a hypertrophic phenotype^35^. Although we did not examine changes in p27^Kip1^ expression during the course of DN, our findings suggest the involvement of p27^Kip1^ transcriptional regulation in LPA-induced proliferation during the initial development of DN.

Among the various transcription factors that regulate p27^Kip1^, KLF5 directly regulates p27^Kip1^ mRNA levels and induces cancer cell proliferation^19,20^. Levels of the KLF5 mRNA and proteins were increased in both the LPA-treated mesangial cells and the kidney cortex of *db/db* mice compared with control mice and cells. In addition, the inhibition of KLF5 expression abolished the pro-proliferative effect of LPA by increasing p27^Kip1^ levels. As shown in the study by Zhang et al., LPA increases colon cancer cell proliferation by upregulating KLF5^11^. Moreover, KLF5 induces cyclin D1 gene expression during the angiotensin II-induced proliferation of vascular smooth muscle cells^36^. Therefore, LPA-induced KLF5 upregulation may directly increase cyclin D1 expression and decrease p27^Kip1^ expression in mesangial cells, leading to hyperproliferation.

The MAPK cascade is an important intracellular signal transduction pathway that contributes to mesangial cell proliferation. MAPK activation has been reported in glomeruli obtained from diabetic animals and mesangial cells under high glucose conditions^37,38^. Furthermore, LPA activated the Erk, JNK, and p38 pathways in the present study. The transcription factor Egr1 has recently been shown to be associated with the MAPK pathway. Egr1 plays a key role in glucose-induced proliferation and renal fibrosis by regulating the expression of extracellular matrix genes in mesangial cells^39,40^. According to Iyoda et al., LPA-induced Egr1 expression in smooth muscle cells is mediated by the activation of Erk1/2 and JNK^23^. In the present study, LPA increased Egr1 expression in mesangial cells, and the effect depended on the activation of the MAPK pathway (p38). Moreover, Egr1 binds to the KLF5 promoter^21^, consistent with our results showing that MAPK activation induced by the LPA treatment increased Egr1 expression and subsequently increased KLF5 expression in mesangial cells.

Rho family small GTPases are important regulators of actin cytoskeletal dynamics, cell motility, and polarity^41^. Although RhoA, Rac, and Cdc42 are required for normal cellular functions, Rac1 overexpression or activation may be involved in the development of renal diseases, including DN. A high-salt diet, angiotensin II, and oxalate upregulate Rac1, leading to renal injury in vivo and in vitro^42,43^. Gumustekin et al. reported higher Rac1 expression in the kidneys of diabetic rats compared with control rats^44^, and Hubchak et al. reported that Rac1 activity increased TGFβ1-induced type 1 collagen expression in mesangial cells^45^. In our study, LPA increased Rac1 activity, but not RhoA and Cdc42 activity, consistent with the results of a previous study showing that Rac1 activation was mediated by LPAR1 signaling^46^. Although we did not observe direct effects of LPAR1 and Rac1 activation, the inhibition of LPAR1 signaling ameliorated DN in diabetic mice in our previous study^14^. Therefore, LPA-induced Rac1 activation was mediated by LPAR1 in mesangial cells.

Inhibition of Rac1 activity reduced KLF5 expression and MAPK (p38) phosphorylation, resulting in delayed LPA-induced hyperproliferation. Lv et al. reported that podocyte-specific Rac1 depletion attenuated diabetic podocyte injury, and p38 phosphorylation mediated this effect^47^. In addition, treatment with a Rac1 inhibitor reduces albumin excretion and glomerular damage in an animal model of chronic kidney disease^48^. Based on these findings, Rac1 activation was one of the mechanisms by which LPA induced hyperproliferation during the initial phase of DN.

**Fig. 7:**
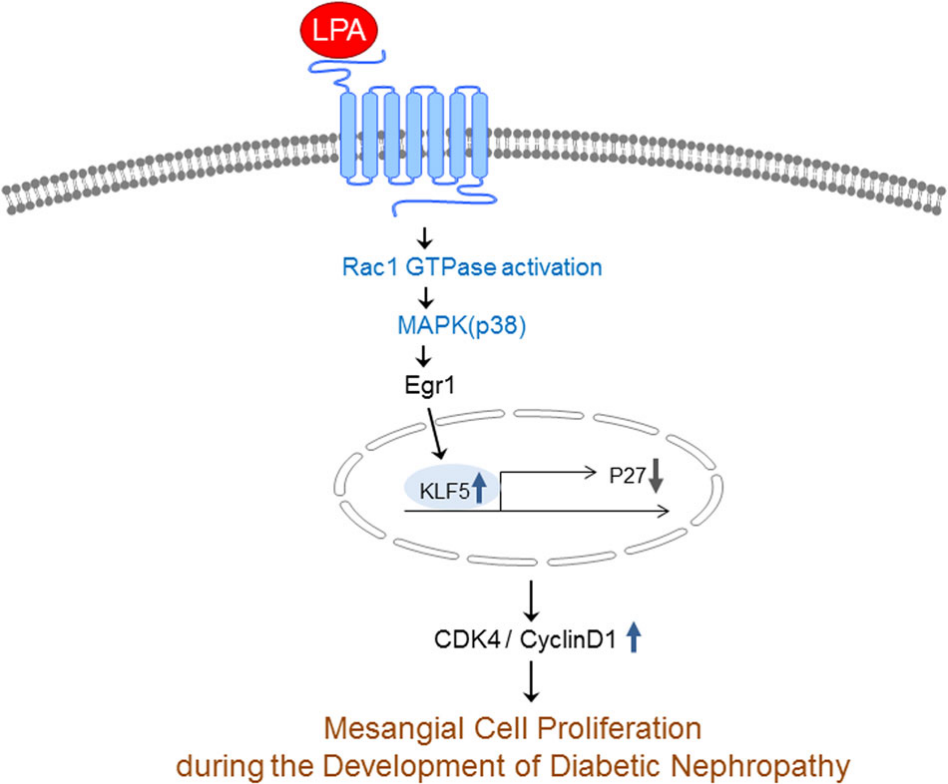
Schematic of the mechanism by which LPA induced mesangial cell proliferation in diabetic nephropathy models through Rac1/MAPK/KLF5 signaling

In summary, our study revealed the pro-proliferative effect of LPA on mesangial cells and the involvement of the Rac1/MAPK/KLF5 signaling pathway (Fig. 7). These pathways contribute to glomerular hyperproliferation during the progression of DN, and strategies regulating these pathways may represent a potential anti-proliferative therapy for DN.

## Supplementary information

Supplementary materials

## References

[1] Himmelfarb J, Tuttle KR (2013). New therapies for diabetic kidney disease. N. Engl. J. Med..

[2] Gonzalez Suarez ML, Thomas DB, Barisoni L, Fornoni A (2013). Diabetic nephropathy: is it time yet for routine kidney biopsy?. World J. Diabetes.

[3] Wolf G (2000). Cell cycle regulation in diabetic nephropathy. Kidney Int. Suppl..

[4] Kanwar YS (2008). Diabetic nephropathy: mechanisms of renal disease progression. Exp. Biol. Med. (Maywood)..

[5] Kolset SO, Reinholt FP, Jenssen T (2012). Diabetic nephropathy and extracellular matrix. J. Histochem. Cytochem..

[6] Schena FP, Gesualdo L (2005). Pathogenetic mechanisms of diabetic nephropathy. J. Am. Soc. Nephrol..

[7] Lin ME, Herr DR, Chun J (2010). Lysophosphatidic acid (LPA) receptors: signaling properties and disease relevance. Prostaglandins Other Lipid Mediat..

[8] Inoue CN (1999). Lysophosphatidic acid and mesangial cells: implications for renal diseases. Clin. Sci. (Lond.).

[9] Xing Y, Ganji SH, Noh JW, Kamanna VS (2004). Cell density-dependent expression of EDG family receptors and mesangial cell proliferation: role in lysophosphatidic acid-mediated cell growth. Am. J. Physiol. Ren. Physiol..

[10] Gaits F, Salles JP, Chap H (1997). Dual effect of lysophosphatidic acid on proliferation of glomerular mesangial cells. Kidney Int..

[11] Zhang H (2007). Lysophosphatidic acid facilitates proliferation of colon cancer cells via induction of Kruppel-like factor 5. J. Biol. Chem..

[12] Grove KJ (2014). Diabetic nephropathy induces alterations in the glomerular and tubule lipid profiles. J. Lipid Res..

[13] Rancoule C (2013). Lysophosphatidic acid impairs glucose homeostasis and inhibits insulin secretion in high-fat diet obese mice. Diabetologia.

[14] Li HY, Oh YS, Choi JW, Jung JY, Jun HS (2017). Blocking lysophosphatidic acid receptor 1 signaling inhibits diabetic nephropathy in db/db mice. Kidney Int..

[15] Sharma K, McCue P, Dunn SR (2003). Diabetic kidney disease in the db/db mouse. Am. J. Physiol. Ren. Physiol..

[16] Breyer MD (2005). Mouse models of diabetic nephropathy. J. Am. Soc. Nephrol..

[17] Marshall CB, Shankland SJ (2006). Cell cycle and glomerular disease: a minireview. Nephron. Exp. Nephrol..

[18] Shankland SJ, Wolf G (2000). Cell cycle regulatory proteins in renal disease: role in hypertrophy, proliferation, and apoptosis. Am. J. Physiol. Ren. Physiol..

[19] Wang C (2015). The interplay between TEAD4 and KLF5 promotes breast cancer partially through inhibiting the transcription of p27Kip1. Oncotarget.

[20] Chen C (2006). KLF5 promotes cell proliferation and tumorigenesis through gene regulation and the TSU-Pr1 human bladder cancer cell line. Int. J. Cancer.

[21] Kawai-Kowase K, Kurabayashi M, Hoshino Y, Ohyama Y, Nagai R (1999). Transcriptional activation of the zinc finger transcription factor BTEB2 gene by Egr-1 through mitogen-activated protein kinase pathways in vascular smooth muscle cells. Circ. Res..

[22] Windischhofer W (2012). LPA-induced suppression of periostin in human osteosarcoma cells is mediated by the LPA(1)/Egr-1 axis. Biochimie.

[23] Iyoda T (2012). Lysophosphatidic acid induces early growth response-1 (Egr-1) protein expression via protein kinase Cdelta-regulated extracellular signal-regulated kinase (ERK) and c-Jun N-terminal kinase (JNK) activation in vascular smooth muscle cells. J. Biol. Chem..

[24] Burridge K, Doughman R (2006). Front and back by Rho and Rac. Nat. Cell Biol..

[25] Burridge K, Wennerberg K (2004). Rho and Rac take center stage. Cell.

[26] Ichinose K, Kawasaki E, Eguchi K (2007). Recent advancement of understanding pathogenesis of type 1 diabetes and potential relevance to diabetic nephropathy. Am. J. Nephrol..

[27] Wolf G, Ziyadeh FN (1999). Molecular mechanisms of diabetic renal hypertrophy. Kidney Int..

[28] Inoue CN (2001). Bimodal effects of platelet-derived growth factor on rat mesangial cell proliferation and death, and the role of lysophosphatidic acid in cell survival. Clin. Sci. (Lond.).

[29] Dong Y, Sui L, Sugimoto K, Tai Y, Tokuda M (2001). Cyclin D1-CDK4 complex, a possible critical factor for cell proliferation and prognosis in laryngeal squamous cell carcinomas. Int. J. Cancer.

[30] Coqueret O (2003). New roles for p21 and p27 cell-cycle inhibitors: a function for each cell compartment?. Trends Cell Biol..

[31] Sherr CJ (2000). The Pezcoller lecture: cancer cell cycles revisited. Cancer Res..

[32] Liu F (2014). The effect of FoxO1 on the proliferation of rat mesangial cells under high glucose conditions. Nephrol. Dial. Transplant..

[33] Wang B (2011). Bufalin inhibits platelet-derived growth factor-BB-induced mesangial cell proliferation through mediating cell cycle progression. Biol. Pharm. Bull..

[34] Kumar D (2010). Heme oxygenase-1 modulates mesangial cell proliferation by p21 Waf1 upregulation. Ren. Fail..

[35] Wolf G (1997). High glucose stimulates expression of p27Kip1 in cultured mouse mesangial cells: relationship to hypertrophy. Am. J. Physiol..

[36] Gao D (2015). Rosiglitzone suppresses angiotensin II-induced production of KLF5 and cell proliferation in rat vascular smooth muscle cells. PLoS One.

[37] Haneda M (1997). Mitogen-activated protein kinase cascade is activated in glomeruli of diabetic rats and glomerular mesangial cells cultured under high glucose conditions. Diabetes.

[38] Haneda M (1997). Activation of mitogen-activated protein kinase cascade in diabetic glomeruli and mesangial cells cultured under high glucose conditions. Kidney Int. Suppl..

[39] Wang D (2015). Transcription factor Egr1 is involved in high glucose-induced proliferation and fibrosis in rat glomerular mesangial cells. Cell. Physiol. Biochem..

[40] Bryant M (2000). Tissue repair with a therapeutic transcription factor. Hum. Gene Ther..

[41] Ridley AJ (2003). Cell migration: integrating signals from front to back. Science.

[42] Shibata S (2011). Rac1 GTPase in rodent kidneys is essential for salt-sensitive hypertension via a mineralocorticoid receptor-dependent pathway. J. Clin. Invest..

[43] Thamilselvan V, Menon M, Thamilselvan S (2012). Selective Rac1 inhibition protects renal tubular epithelial cells from oxalate-induced NADPH oxidase-mediated oxidative cell injury. Urol. Res..

[44] Gumustekin M (2013). The effect of insulin treatment on Rac1 expression in diabetic kidney. Ren. Fail..

[45] Hubchak SC, Sparks EE, Hayashida T, Schnaper HW (2009). Rac1 promotes TGF-beta-stimulated mesangial cell type I collagen expression through a PI3K/Akt-dependent mechanism. Am. J. Physiol. Ren. Physiol..

[46] Van Leeuwen FN (2003). Rac activation by lysophosphatidic acid LPA1 receptors through the guanine nucleotide exchange factor Tiam1. J. Biol. Chem..

[47] Lv Z (2018). Podocyte-specific Rac1 deficiency ameliorates podocyte damage and proteinuria in STZ-induced diabetic nephropathy in mice. Cell Death Dis..

[48] Babelova A (2013). Activation of Rac-1 and RhoA contributes to podocyte injury in chronic kidney disease. PLoS One.

