# Supplementary material for: Lysophosphatidic acid increases mesangial cell proliferation in models of diabetic nephropathy via Rac1/MAPK/KLF5 signaling
Source: Exp Mol Med. 2019 Feb 15;51(2):18. doi: 10.1038/s12276-019-0217-3 (PMC6377648; doi:10.1038/s12276-019-0217-3)
Supplement: Supplementary file 1 — Supplementary materials [file 12276_2019_217_MOESM1_ESM.docx]

**Supplementary Materials**

***Quantitative real time PCR (qRT-PCR)***

Total RNA was prepared using RNAiso Plus and cDNA was synthesized as described in Materials and Methods. Primers used for qRT-PCR are listed in Supplementary table 1.

***Supplementary Table 1. Primers for qRT-PCR analysis.***

| Gene | Forward | Reverse |
| --- | --- | --- |
| cyclophilin | TGGAGAGCACCAAGACAGACA | TGCCGGAGTCGACAATGAT |
| p15^INK4b^ | AGATCCCAACGCCCTGAAC | TGCCGGAGTCGACAATGAT |
| p16^INK4a^ | CGCCCCGAACTCTTTCG | GGGTTGTGAATCGCCAGTTT |
| p21^Cip1^ | GAATCTTCAGGCCGCTCAGA | CCCGTTGCTCAGCAGCAT |
| p27^Kip1^ | TCTTCGGCCCGGTCAA | CCGGCAGTGCTTCTCCAA |

**Supplementary Figure 1**

**
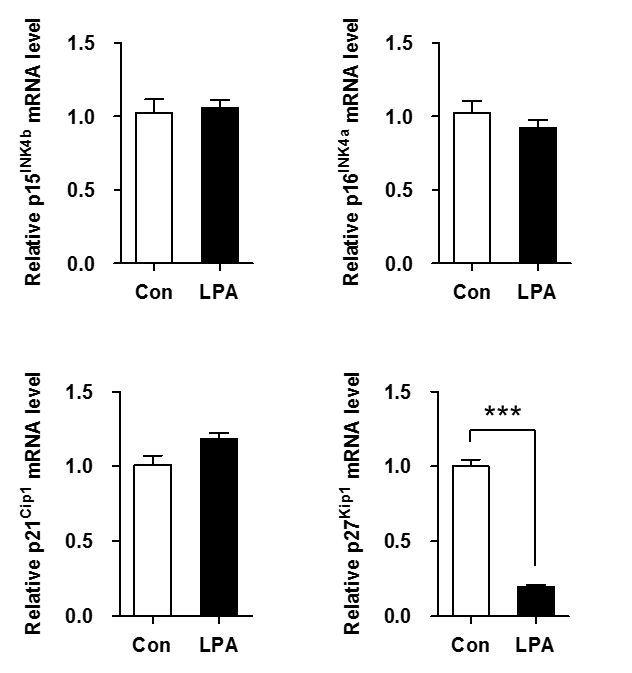
**

**Supplementary Figure 1. LPA decreases the mRNA expression of p27^Kip1^, but not p15^INK4b^, p16^INK4a^, and p21^Cip1^.** SV40 MES13 cells were seeded in a 6-well plate and starved in serum-free medium containing 0.1% fatty acid-free bovine serum albumin for 12–16 h. Cells were treated with LPA (10 μM) for 6 h, and the mRNA levels of p15^INK4b^, p16^INK4a^, p21^Cip1^, and p27^Kip1^ were analyzed by qRT-PCR (n=3~4 independent experiments). ***p<0.001. Data represent the mean ± SEM.

**Supplementary Figure 2**


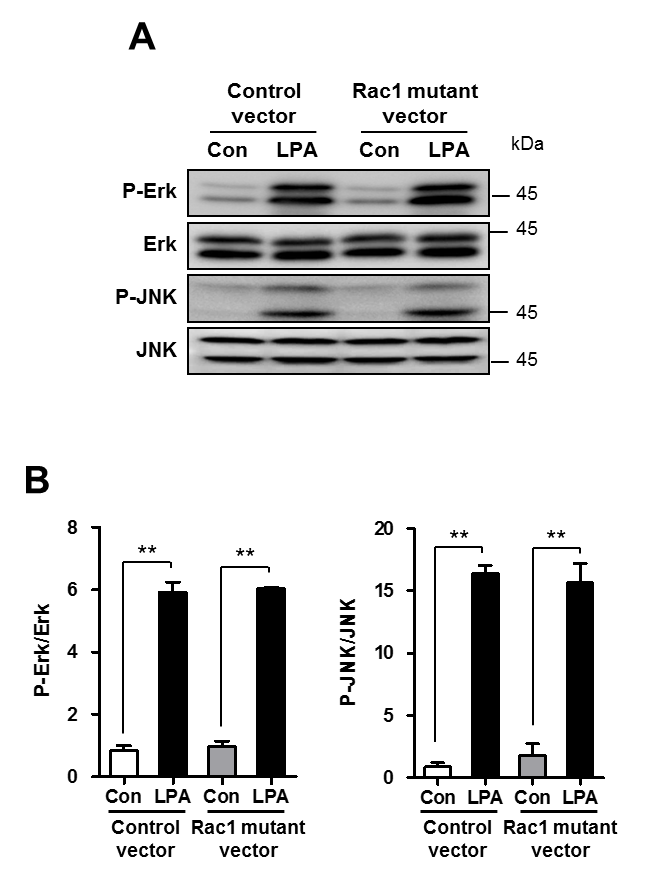


**Supplementary Figure 2. LPA activates Erk and JNK through a Rho GTPase Rac1-independent pathway in SV40 MES13 cells.** After the transfection of SV40 MES13 cells with the control vector or dominant-negative Rac1 mutant vector, cells were starved in serum-free medium containing 0.1% fatty acid-free bovine serum albumin for 12–16 h. Then, cells were treated with LPA (10 μM) for 15 min. **(A)** The protein levels of P-Erk, Erk, P-JNK, and JNK were determined by western blot analysis. **(B)** The relative expressions of P-Erk, and P-JNK were normalized to that of Erk or JNK, and quantified using the ImageJ software (n=3 independent experiments). **p<0.01. Data represent the mean ± SEM.
